# Reducing Xerostomia by Comprehensive Protection of Salivary Glands in Intensity-Modulated Radiation Therapy with Helical Tomotherapy Technique for Head-and-Neck Cancer Patients: A Prospective Observational Study

**DOI:** 10.1155/2019/2401743

**Published:** 2019-07-14

**Authors:** Feng Teng, Wenjun Fan, Yanrong Luo, Zhongjian Ju, Hanshun Gong, Ruigang Ge, Fang Tong, Xinxin Zhang, Lin Ma

**Affiliations:** ^1^Medical School of Chinese PLA, 28 Fuxing Road, Beijing 100853, China; ^2^Department of Radiation Oncology, China-Japan Friendship Hospital, 2 Yinghuayuan Dongjie, Beijing 100029, China; ^3^Armed Police Corps Hospital of Henan Province, Zhengzhou 450052, China

## Abstract

**Objective:**

This study aimed to analyze the effects of comprehensive protection of bilateral parotid glands (PG-T), contralateral submandibular gland (cSMG), and accessory salivary glands in the oral cavity (OC) by helical tomotherapy for head-and-neck cancer patients.

**Methods:**

Totally 175 patients with histologically confirmed head-and-neck cancer treated with helical tomotherapy were recruited. The doses delivered to PG-T, cSMG, and OC were constrained to be as low as possible in treatment planning. The saliva flow rates and xerostomia questionnaire were evaluated. Correlation between xerostomia and other clinical factors were assessed using univariate and multivariate models. The impact of salivary gland dose on locoregional (LR) recurrence was assessed by Cox analysis. ROC curve was used to determine the threshold of mean dose for each gland.

**Results:**

The median follow-up was 25 (19–36) months. The OC mean dose, PG-T mean dose, cSMG mean dose, age, clinical stage (II and III versus IV), and both unstimulated and stimulated saliva flow rates were significantly correlated with xerostomia. The OC mean dose, cSMG mean dose, age, and clinical stage were predictors of xerostomia after adjusting PG-T mean dose, and unstimulated and stimulated saliva flow rates. Xerostomia was significantly decreased when the mean doses of PG-T, cSMG, and OC were kept below 29.12Gy, 29.29Gy, and 31.44Gy, respectively. At 18 months after radiation therapy, early LR recurrence rate was only 4%.

**Conclusion:**

Comprehensive protection of salivary glands minimized xerostomia in head-and-neck cancer patients treated by helical tomotherapy, without increasing early LR recurrence risk.

## 1. Background

The overall incidence of head-and-neck cancer accounted for 5%~10% of the total body malignant tumors [1]. Intensity-modulated radiation therapy (IMRT) is one of the main methods for the treatment of head-and-neck cancer, but radiation-induced xerostomia caused due to decreased salivary gland function is one of the most common and serious adverse effects. Xerostomia seriously impacts the quality of life of the patient, causing difficulties in chewing, swallowing, speaking, sleeping, secondary oral infection, radioactive caries, and other diseases [2, 3]. Therefore, in order to alleviate xerostomia and improve the quality of life, it is necessary to protect the salivary glands during radiotherapy of head-and-neck cancer.

The parotid gland (PG) is the largest salivary gland and is the main source of stimulated saliva [4]. In order to relieve from dry mouth, some researchers reduced the volume and dose of radiation to the parotid glands by IMRT [5–10]. Although IMRT protects the parotid glands, the symptoms of dry mouth are still obvious in some patients. The reason for this is that the other salivary glands present beside the parotids are also exposed to high doses of radiation, such as submandibular glands, sublingual glands, and many small salivary glands. The submandibular gland (SMG) produces about 70% of unstimulated saliva that accounted for over 90% of saliva secretion at night. Many authors reported that xerostomia could be alleviated after SMG-sparing IMRT [11–14]. However, the submandibular gland is adjacent to IB and II lymph nodes. Currently, there is no clinical trial with large sample size to determine whether the protection of the submandibular gland leads to increased recurrence rate of adjacent lymph nodes. In addition, with the sublingual gland, small salivary glands are widely distributed throughout the oral mucosa and secrete more than 70% of the mucin in saliva. This plays an important role in the maintenance of lubrication comfort of the oral mucosa [15–17]. Chajon et al. [18] reported that the locoregional (LR) recurrence risk was not increased when using IMRT with a whole salivary gland-sparing strategy, including PG, SMG, and oral cavity (OC). At present, it is unclear whether comprehensive protection of salivary glands during IMRT by helical tomotherapy technique for head-and-neck cancer patients can significantly reduce xerostomia without increasing the LR recurrence risk. Hence, this prospective observational study was conducted. Objective measurement of saliva flow rate and subjective xerostomia questionnaire evaluation are used to evaluate the improvement of oral dryness.

## 2. Materials and Methods

### 2.1. Patients

Between February 2016 and July 2017, 176 patients with histologically confirmed squamous cell carcinoma of head and neck, treated by helical tomotherapy technique, were collected at Radiotherapy Department of the General Hospital of the Chinese People's Liberation Army. One patient who had Sjogren's syndrome was excluded from analysis. The clinical characteristics of the remaining 175 patients are shown in Table 1. All patients were evaluated by MRI or PET-CT for clinical staging according to the International Union Against Cancer (UICC, 2010). These patients had no history of other rheumatic immune system diseases such as Sjogren's syndrome, head-and-neck surgery trauma, or radiotherapy. All the patients signed a written informed consent. This study was conducted in our center and approved (approved no. S2016-122-01) by the Ethics Committee of The General Hospital of the Chinese People's Liberation Army, registered with number ChiCTR-ONN-17010597 in Chinese Clinical Trial Registry.

### 2.2. Radiation Therapy

The patients were placed in supine position and fixed with thermoplastic film for head-neck-shoulder. Simulated CT enhanced scan with 3.0mm slice thickness was acquired. Imaging must clearly show the extent of tumor lesion invasion, lymph node metastasis, and the structure of parotid glands, submandibular glands, and oral cavity. The multimode image fusion technology was used as a reference to delineate the target area and the organs at risk. The delineation standards were described according to the ICRU report 83 and combined with the department's treatment specifications. The delineation of the target area was accurate, and the target area was not compromised with the relevant salivary gland protection. Irradiation with daily image guidance was performed using 6MV photon beam obtained from TomoTherapy (Accuray, USA).

### 2.3. IMRT, Dose Prescription, and Plan Evaluation

The radiotherapy plan was optimized on the Pinnacle 3 8.0 workstation by reverse intensity-modulated planning system and was evaluated by dose volume histograms (DVHs). The physiologists and clinicians participated in the evaluation, and the relevant parameters were as follows: the prescribed dose was required to include at least 95% of the target volume. The prescribed doses were as follows: gross target volume of the primary tumor (GTVnx) = metastatic lymph node lesions (GTVnd) = 67.5-70Gy/30-33f, high-risk clinical target volume (CTV1)= 60 Gy, and low-risk clinical target volume (CTV2) = 54 Gy. Gland dose limitation should be as low as possible for PG, SMG, and OC (with small salivary glands). RTOG standard was adopted for evaluating adverse reactions.

### 2.4. Xerostomia and the Salivary Gland Function Assessment

Xerostomia was evaluated by a questionnaire at 0, 1, 3, 6, 12, and 18 months after the end of radiotherapy. The xerostomia questionnaire (XQ) (Table 2) was modified based on the questionnaire tested and validated by Amosson et al. [19]. This consisted of 10 questions in this scale, involving the patient's diet, chewing, swallowing, speaking, sleeping, drinking, and other aspects. Each question was divided into four grades according to the degree of patient's reported mouth dryness, with “no,” “mild,” “moderate,” and “severe.” Each grade was assigned a score of 0, 1, 2, and 3. According to the investigation in patients, the total score was calculated and the degree of mouth dryness was classified; i.e., the higher the value, the more serious the xerostomia. The xerostomia classification was as follows: mild dry mouth: total score of ≤10 points; moderate dry mouth: 10 points≤ total points < 20 points; and severe dry mouth: total ≥20 points.

The total salivary secretion was measured before the start of radiotherapy and at 0, 1, 3, 6, 12, and 18 months after the end of radiotherapy. The patients were advised not to eat and drink for 1 hour and then sit on a chair by supporting both the elbows on both the knees, with holding funnel (connected to the centrifugal tube) in the hands, head as low as possible, eyes opened, funnel back edge against the patient's lip and cheek angle, and the tip of the tongue against the upper jaw. The unstimulated saliva flow rates were measured simultaneously for 5 min with the patient spitting all saliva into the funnel and followed by the collection of stimulated saliva flow for further 5 min with stimulation by applying 2% citric acid solution to the tongue [20].

### 2.5. Statistical Methods

The continuous variables were expressed as mean ± standard deviation or as median with interquartile range according to the normal or skewed distribution. Statistical comparisons of continuous variables were performed using independent samples* t* test or Mann–Whitney U test for the two groups. Categorical variables were expressed as percentages. Statistical comparisons between the two groups were performed using *χ*2 test or Fisher's exact test. An ROC curve analysis was applied to detect the cut-off point related to the salivary glands mean dose by utilizing the maximal Youden index method. We also performed a multivariable linear regression analysis to detect the factors of patient-reported xerostomia scores. All statistical tests were performed by IBM SPSS 25.0 statistical software, and a two-sided p< 0.05 was deemed to be statistically significant.

## 3. Results

### 3.1. Patients

Between February 2016 and July 2017, a total of 175 patients were enrolled in this study. Patient baseline characteristics are presented in Table 1. Patients were predominantly male (73.7%), with a median age of 49 years (range from 11 to 83 years). The patients had nasopharyngeal cancer (73.14%), hypopharyngeal cancer (11.43%), oropharyngeal cancer (2.86%), oral cavity cancer (5.14%), laryngeal cancer (4.0%), nasal cavity and paranasal sinuses cancer (2.29%), and other cancers (1.14%), with stages II (13.14%), III (34.29%), and IV (52.57%). One hundred and sixteen patients (66.3%) received induction chemotherapy combined with concurrent chemoradiotherapy. Fifty-four patients (30.9%) received induction chemotherapy combined with concurrent chemoradiotherapy and molecular targeted therapy. The median time from therapy to last follow-up visit was 25 months (19–36 months).

### 3.2. Salivary Gland Function following IMRT by Helical Tomotherapy Technique

The doses were constrained to be as low as possible following IMRT by helical tomotherapy technique for bilateral PG (PG-T, with the average doses of both glands), contralateral SMG (cSMG), and OC, with an average of the mean dose of these glands of 29.50Gy (range from 11.19 to 46.53Gy), 31.03Gy (range from 10.29 to 49.26Gy), and 31.53Gy (range from 13.55 to 54.10Gy), respectively. Saliva flow rate and xerostomia questionnaire (XQ) were used to evaluate the improvement of mouth dryness.

### 3.3. Saliva Flow Rates

**Figure 1:**
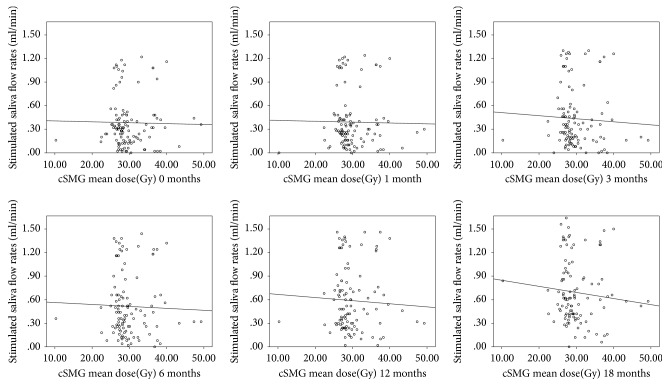
Plots of unstimulated saliva flow rates versus cSMG mean dose at different postradiotherapy time points (0, 1, 3, 6, 12, and 18 months).

**Figure 2:**
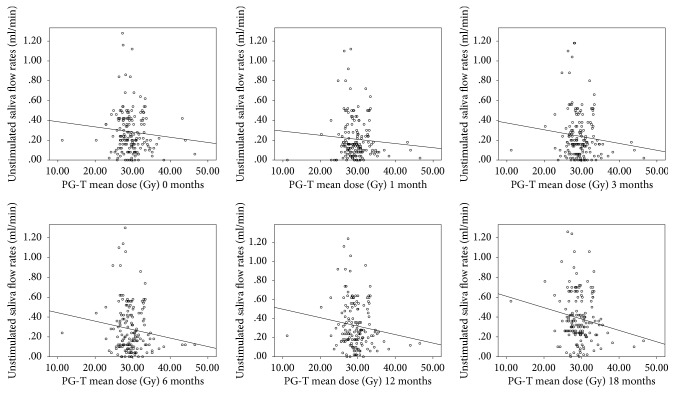
Plots of stimulated saliva flow rate versus PG-T mean dose at different postradiotherapy time points (0, 1, 3, 6, 12, and 18 months).

**Figure 3:**
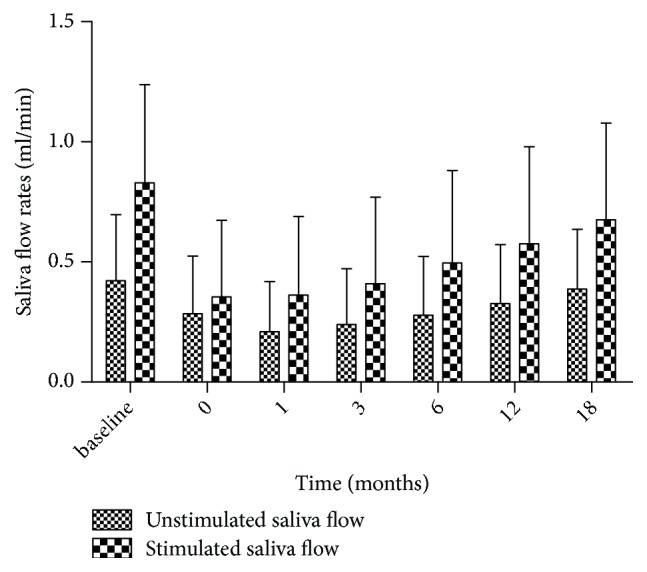
Unstimulated saliva flow rates versus stimulated saliva flow rates at different postradiotherapy time points (0, 1, 3, 6, 12, and 18 months). (Vertical bars represent standard deviation.)

A significant interaction between stimulated saliva flow rate and mean dose of the PG-T was observed, with an interaction between unstimulated saliva flow rate and mean dose of the cSMG. Plots for stimulated and unstimulated flow rates at each postradiotherapy time point are provided in Figures 1 and 2. These figures showed that the mean dose of PG-T or cSMG for most of the patients was more or less than 30 Gy. In addition, the saliva flow rates were decreased gradually with increased mean dose.  Figure 3 showed that the unstimulated and stimulated flow rates decreased initially and then increased with prolonged follow-up. The stimulated/unstimulated saliva flow rates restored to 69.5%/77.4% of the baseline at 12 months and 81.5%/91.7% at 18 months, respectively.

### 3.4. Xerostomia Questionnaire

The XQ scores were available for 100%, 97.1%, 97.1%, 94.9%, 94.9%, and 94.9% of patients at 0th, 1st, 3rd, 6th, 12th, and 18th month, respectively. The factors correlated with patient-reported xerostomia (at 18th month) detected by univariate analysis are summarized in Table 3. The OC mean dose, PG-T mean dose, cSMG mean dose, age, clinical stage (II and III versus IV), and both stimulated and unstimulated saliva flow rates were each statistically significant correlates in the univariate analysis, while the gender was not significant. Univariate analyses of significant variables (P < 0.05) were included in the multivariate analysis. Based on the uncertain effect of gender on patients' reported xerostomia and the unbalanced gender ratio in the enrolled patients (73.7% male versus 26.3% female), gender was also included in the multivariate analysis in this study. The OC mean dose, cSMG mean dose, age, and clinical stage were predictors of patient-reported xerostomia after adjusting for PG-T mean dose, as well as both stimulated and unstimulated saliva flow rates (Table 3). The findings of multivariate analysis showed that the OC mean dose, cSMG mean dose, age, and tumor stage were important predictors of patient-reported xerostomia even after considering the effect of PG mean dose. Mouth dryness restored more slowly with increasing age. In young patients with age ≤35 years (23.67% of patients), xerostomia has been restored nearly to normal level within one year after radiotherapy.

**Figure 4:**
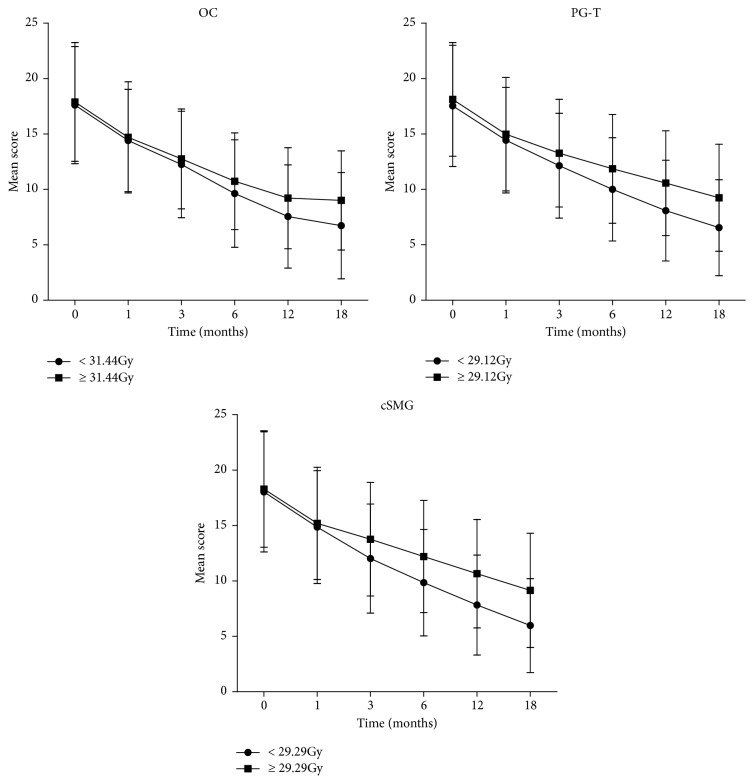
Xerostomia questionnaire (XQ) score variation in patients with mean dose higher or lower than D50. (Vertical bars represent standard deviation.)

In addition, ROC curves were used to estimate the threshold D50 at which the dry symptoms were relieved above 50% at 1 year compared to the end of radiotherapy. The D50 of the mean doses of PG-T, cSMG, and OC were 29.12Gy, 29.29Gy, and 31.44Gy, respectively, and an increased effect on xerostomia restoration over time was observed (Figure 4). As shown in Figure 4 lower D50 of mean dose for each salivary structure was associated with lower (better) XQ scores at each time point during the follow-up period. The XQ score variation showed no statistical difference in patients within 3 months after receiving PG-T mean doses higher or lower than 29.12 Gy; however it varied significantly at 6, 12, and 18 months after radiotherapy (p = 0.011, 0.001, and <0.001, respectively). Similarly, the XQ score varied significantly at 6, 12, and 18 months after radiotherapy in patients with the cSMG mean doses higher or lower than 29.29 Gy (p = 0.016, 0.003, and 0.001, respectively). XQ score also showed a significant difference in patients with the OC mean dose higher or lower than 29.12 Gy at 18 months (p = 0.002).

### 3.5. Comprehensive Protection of Salivary Glands and LR Recurrence Risk

We observed that the comprehensive protection of salivary glands significantly reduced the risk of developing of severe xerostomia, without a compromised locoregional control, as the locoregional recurrence rate was only 4%, with 6 recurrences at the primary tumor site and only 1 recurrence at level II of nodes. In this study, 61.3% of patients with xerostomia restored to normal level at 1 year, and 69.6% at 18 months after radiotherapy. The 18-month locoregional relapse-free survival was 86.3%.

## 4. Discussion

As known to all, multiple studies showed that IMRT can decrease the radiation-related xerostomia by sparing the salivary glands without increasing the locoregional recurrence risk. Helical tomotherapy, computed tomography-based platform for IMRT, provides superior homogeneous covering of the target volume, while delivering the lowest doses to the salivary glands. This study was the first in sparing all the salivary glands during IMRT by helical tomotherapy technique for head-and-neck cancer, demonstrating that when dose was constrained to the parotid gland, submandibular gland, and oral cavity, 61.3% and 69.6% of xerostomia patients restored to normal level at 12 and 18 months after radiotherapy, respectively, with an early locoregional recurrence rate of 4%.

The major glands (PG, SMG, and OC) produce above 90% of saliva [17]. The PG is the main source of stimulated saliva, while the SMG is the main source of unstimulated saliva. The PG is radiation-sensitive. For example, a dose of 15~20Gy can significantly reduce the amount of saliva and cause dry mouth symptoms, while 40~50Gy dose can cause permanent functional damage of PG. So, higher irradiation doses can cause irreversible damage to the glands, and then dry mouth symptoms are more severe and persistent and even permanent [5]. Lin et al. [6] found that xerostomia and quality of life were significantly improved by IMRT by protection of PG in 36 patients after radiotherapy at 1 year by following xerostomia questionnaire (XQ) and quality of life survey (QOL). Tribius et al. [21] suggested that sparing both PGs compared with one PG (left or right) can reduce xerostomia without compromising survival. In this study, there was no significant difference in the mean dose received by left (29.59 Gy, 17.68-58.74 Gy) versus right (29.41 Gy, 4.69-62.86 Gy) PG. For healthy PG, it is generally assumed that a homogenous distribution of saliva production takes place over the entire volume, and the mean of both PGs was used to assess the exposed dose in PG [22, 23]. Saarilahti et al. [14] concluded that sparing of cSMG resulted in a substantially better reduction of xerostomia as compared to patients with only one parotid gland spared. In our study, xerostomia was not associated with LR recurrence within the spared cSMG area. SMG-sparing IMRT realized by helical tomotherapy technique is an effective method to reduce the risk of xerostomia in head-and-neck cancer patients. Also, the OC mean doses showed significant effects on mouth dryness after adjusting for PG-T and cSMG mean doses. The results justified efforts to spare all the salivary glands, beyond the PGs alone.

The D50 doses of all the glands (PG-T, cSMG, and OC) were 29.12Gy, 29.29Gy, and 31.44Gy, respectively. The D50 dose is the gland dose at which the dry symptoms were relieved by above 50% at 1 year compared to the end of radiotherapy. The use of a D50 dose as a threshold for the control of gland dose remained helpful to evaluate the xerostomia. Kwong et al. [7] studied 33 cases of early nasopharyngeal carcinoma (NPC) and found that the mean parotid threshold dose (median dose (Dmean)) was 38.8gy (32-46.1 Gy) with a relatively good recovery after 2 years of radiotherapy in both saliva flow rate and dry mouth symptoms. Lee et al. [8] studied 67 cases of locally advanced NPC. The parotid D50 acuities in these were found to be 33.2 Gy (left) or 34.2 Gy (right) 2 years after treatment in patients with dry mouth symptoms was more than grade II. In this study, we showed that most of the glands received more or less than 29 Gy, and the stimulated or unstimulated saliva flow rates were decreased gradually with increased mean dose for PG-T or cSMG. It becomes clear that there is an exponential relation between salivary gland function and mean dose for each gland, suggesting that it is essential to have a certain threshold for mean dose to preserve the gland function. We used ROC curves to determine the threshold mean dose for each gland. The D50 of the mean doses of PG-T, cSMG, and OC were 29.12Gy, 29.29Gy, and 31.44Gy, respectively. With lower mean dose than the threshold of D50, the xerostomia score reduced with prolonged follow-up time and showed marked differences at 12 and 18 months after radiotherapy.

Univariate analysis identified some independent influential factors of xerostomia, including salivary glands mean dose (OC, cSMG, and PG-T), age, clinical stage, and both stimulated and unstimulated saliva flow rates. However multivariate analysis implied that, once adjusted for PG-T doses, gender, and both stimulated and unstimulated saliva flow rates, only the OC and cSMG mean doses, age, and clinical stage were important predictors of patient-reported xerostomia. As showed in other studies on the correlation between the salivary glands does and xerostomia, the lack of statistical significance in the multivariate model should not be interpreted to mean a lack of a causal relationship; the reason may be the difficulties in interpreting such results in a regression model with intercorrelating predictors [24, 25]. The XQ could assess the degree of mouth dryness remission paralleled to the saliva flow rates. In addition, usually, the saliva is collected selectively from each major gland. Collection can be either unstimulated or stimulated. Saliva production by all of the glands was collectively measured by spitting into the mouth. The PG is the main source of stimulated saliva, while the submandibular is the main source of unstimulated saliva. However, the results are not always comparable between the salivary flow rates and gland contribution, probably due to the variation in normal salivary flow rates and discrepancies among each gland. This seriously impeded the definition of a threshold of saliva flow rates to evaluate xerostomia.

This study showed that comprehensive protection of salivary glands significantly reduced the risk of developing severe xerostomia. However, our study has some limitations that need to be acknowledged. The follow-up time was too short to allow an accurate assessment of long-term follow-up of dry mouth improvement and locoregional recurrence. The analysis of long-term consequences of glands sparing in these patients is ongoing.

## 5. Conclusions

The comprehensive protection of salivary glands minimized radiation-related xerostomia in head-and-neck cancer patients treated by helical tomotherapy without increasing the locoregional recurrence risk. Xerostomia was significantly decreased when the threshold of mean dose of each gland (PG-T, cSMG, and OC) was maintained below 29.12Gy, 29.29Gy, and 31.44Gy, respectively.

## Figures and Tables

**Table 1:** Patients' characteristics.

Characteristics	No. of patients	%
Age (median range)	49	11-83
Gender		
Male	129	73.7
Female	46	26.3
WHO performance status		
0	79	45.1
1	96	54.9
Treatment		
Induction chemotherapy+	116	66.3
concurrent chemoradiotherapy		
Induction chemotherapy+	54	30.9
concurrent chemoradiotherapy+		
molecular targeted therapy		
Concurrent chemoradiotherapy+	1	0.6
molecular targeted therapy		
Concurrent chemoradiotherapy	2	1.1
Radiotherapy alone	2	1.1
Tumor site		
Oropharynx	5	2.86
Nasopharynx	128	73.14
Hypopharynx	20	11.43
Larynx	7	4.00
Oral cavity	9	5.14
Nasal cavity and paranasal sinuses	4	2.29
Others	2	1.14
T-stage		
T1	17	9.7
T2	61	34.9
T3	37	21.1
T4	38	21.7
T4a	14	8.0
T4b	8	4.6
N-stage		
N0	26	14.9
N1	34	19.4
N2	95	54.3
N3	20	11.4
UICC/AJCC (2010)		
II	23	13.14
III	60	34.29
IVa	65	37.14
IVb	27	15.43

**Table 2:** Xerostomia questionnaire.

1. How is the overall comfort of your mouth?
A very comfortable B mild dryness C moderate dryness D severe dryness
2.Do you feel dryness when eating?
A never B mild (no significant change in feeding habits) C moderate (fluid intake or semi-fluid intake) D severe (requiring nasal feeding tube or intravenous nutrition)
Do you have difficulty swallowing because of dry mouth
A never B mild C moderate D severe
4.Do you have difficulty chewing because of dry mouth?
A never B mild C moderate D severe
5.Do you have problems with speech because of dry mouth?
A never B mild C moderate D severe
6.Do you have problems with sleeping because of dry mouth?
A never B mild C moderate D severe
7.Do you need to drink water when swallowing dry food?
A never B occasionally C frequently D always
8.How often do you need to drink water during the day to keep your mouth comfortable?
A < 1 time/hour B once/hour C 2-3 times/hour D > 3 times/hour
9.How much saliva do you feel in your mouth?
A much B moderate C little D none
10. Has your taste changed?
A never B mild C moderate D severe

**Table 3:** Predictors of patient-reported xerostomia scores.

Variable	Univariate analysis			Multivariate model		
	Estimate	SE	P	Estimate	SE	P
OC Dmean	0.216	0.074	0.004	0.226	0.091	0.014
PG-T Dmean	0.226	0.098	0.023	-0.023	0.117	0.844
cSMG Dmean	0.317	0.089	0.001	0.183	0.089	0.043
Age	0.125	0.024	<0.001	0.092	0.035	0.009
Stage (II-III vs. IV)	1.494	0.728	0.042	1.842	0.850	0.033
Gender	-1.323	0.832	0.114	-1.875	0.990	0.061
Unstimulated saliva flow rate	-4.632	1.497	0.002	-2.181	2.351	0.356
Stimulated saliva flow rate	-4.113	0.899	<0.001	-1.892	1.465	0.200

*∗*Dmean: mean dose.

## Data Availability

The datasets used and analysed during the current study are available from the corresponding author on reasonable request.
